# Nucleosome positioning and kinetics near transcription-start-site barriers are controlled by interplay between active remodeling and DNA sequence

**DOI:** 10.1093/nar/gkt854

**Published:** 2013-09-24

**Authors:** Jyotsana J. Parmar, John F. Marko, Ranjith Padinhateeri

**Affiliations:** ^1^Department of Biosciences and Bioengineering, Indian Institute of Technology Bombay, Mumbai 400076, India, ^2^Department of Molecular Biosciences, Northwestern University, Evanston, IL 60208, USA, ^3^Department of Physics and Astronomy, Northwestern University, Evanston, IL 60208, USA and ^4^Wadhwani Research Centre for Biosciences and Bioengineering, Indian Institute of Technology Bombay, Mumbai 400076, India

## Abstract

We investigate how DNA sequence, ATP-dependent chromatin remodeling and nucleosome-depleted ‘barriers’ co-operate to determine the kinetics of nucleosome organization, in a stochastic model of nucleosome positioning and dynamics. We find that ‘statistical’ positioning of nucleosomes against ‘barriers’, hypothesized to control chromatin structure near transcription start sites, requires active remodeling and therefore cannot be described using equilibrium statistical mechanics. We show that, unlike steady-state occupancy, DNA site exposure kinetics near a barrier is dominated by DNA sequence rather than by proximity to the barrier itself. The timescale for formation of positioning patterns near barriers is proportional to the timescale for active nucleosome eviction. We also show that there are strong gene-to-gene variations in nucleosome positioning near barriers, which are eliminated by averaging over many genes. Our results suggest that measurement of nucleosome kinetics can reveal information about sequence-dependent regulation that is not apparent in steady-state nucleosome occupancy.

## INTRODUCTION

Chromatin consists of nucleosomes bound to DNA in patterns correlated with DNA primary sequence (1–4). Current ‘nucleosome sequencing’ experiments indicate a great deal of heterogeneity of nucleosome positioning, with some regions of precise nucleosome occupancy and other regions that are apparently much less well ordered. The origin of nucleosome ‘positioning’ along DNA remains controversial: some researchers stress the role of the DNA-sequence-dependence of histone–DNA interactions (1–3), whereas others emphasize roles of other mechanisms to control positions of nucleosomes, notably ‘statistical’ positioning of nucleosomes (5–8).

Statistical positioning follows from the existence of ‘barriers’ to nucleosome formation, i.e. locations along DNA that nucleosomes are unable to occupy. For example, non-histone proteins bound strongly to a specific DNA site might sterically prevent nucleosomes from occupying that location. The correlations in nucleosome positions near such a barrier can generate spatial variations of nucleosome occupancy, but without the necessity of any intrinsic DNA-sequence-dependence of histone–DNA interactions (5). Statistical positioning near barriers has been suggested as the origin of nucleosome positioning patterns near transcription start sites (TSS) (6,7,9–11), but without mechanistic understanding of the origin of the barriers. However, all theoretical analyses of statistical positioning have been based on ‘thermal’ equilibration of nucleosome positions along DNA (12–14).

The rates at which nucleosomes can become positioned are crucially dependent on the kinetics of nucleosome placement, relocation and eviction. Given the 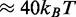 free energy associated with histone–DNA interactions in a nucleosome (15,16), eviction requires non-thermal processes. Efficient nucleosome relocation (‘sliding’) also requires non-thermal kinetics due to the very slow thermal diffusion of nucleosomes along DNA (17–19). This casts considerable doubt on the relevance of equilibrium-statistical-mechanical descriptions of nucleosome positioning (20). Indeed, prior work has shown that assembly of chromatin with nucleosomes spaced by 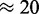 bp linker DNAs on biologically relevant timescales requires active (non-thermal, e.g. ATP-powered) chromatin remodeling. In the absence of active remodeling, nucleosomes cannot reach the degree of packing and positioning observed *in vivo* (17).

In accord with this, Zhang *et al.* (7) have observed experimentally that the apparent statistical positioning observed near TSS barriers requires ATP, presumably to facilitate chromatin remodeling. However, at the same time, other experiments suggest that DNA sequence does play a role in positioning nucleosomes near TSS (1,10,21). These observations suggest that the organization of nucleosomes near TSS is determined by interplay between primary DNA sequence-dependent nucleosome positioning and statistical positioning near nucleosome barriers, but driven by non-thermal ATP-dependent chromatin remodeling dynamics outside of the realm of description in terms of free energies and the (thermal equilibrium) Boltzmann distribution.

Prior theoretical studies on nucleosome positioning near TSSs suggest that DNA sequence is not a crucial factor in reproducing experimentally observed nucleosome occupancy (5,13). However, a number of recent experiments indicate that sequence-dependent nucleosome stability near TSSs has an important role in a range of biological functions (21–23). This leads to an apparent paradox: sequence appears to influence nucleosome stability and biological function, but not occupancy.

We can anticipate that, owing to ATP-driven remodeling, nucleosome organization is highly dynamic, with kinetics and time-averaged properties which are far from thermodynamic equilibrium. These non-thermal nucleosome dynamics likely control site accessibility of DNA binding sites for site-specific DNA-binding proteins, perhaps most notably near TSSs. Given our previously developed model for chromatin dynamics with sequence-dependent nucleosome–DNA interactions and ATP-dependent remodeling (17), we decided to analyze what the dynamics of nucleosomes would be near to nucleosome-depleted barriers.

Here we develop a theoretical description of nucleosome dynamics near barriers where we can examine the effect of sequence as well as ATP-dependent remodeling. We find that establishment of apparent statistical positioning on biologically relevant timescales requires active chromatin remodeling; statistical positioning cannot occur by the action of thermal fluctuations alone, in accord with the result of Zhang *et al.* (7). Furthermore, we find that DNA sequence does control nucleosome occupancy relatively near to TSS barriers, but that this effect is suppressed when one averages occupancy over many genes. Computing nucleosome assembly kinetics, we show that the timescale for formation of statistical positioning is proportional to the timescale of active nucleosome disassembly. Finally, we also observe that the kinetics of site exposure show strong sequence dependence adjacent to nucleosome barriers, indicating that there may be strong effects of DNA-sequence-dependent nucleosome binding on, for example, the kinetics of gene regulation.

The calculations of this article are all applied to chromatin dynamics of *Saccharomyces cerevisiae*, for which detailed genome-wide nucleosome positioning and remodeling enzyme kinetic data exist. However, owing to the generic nature of our model, we anticipate that our results should be at least qualitatively relevant to chromatin dynamics across a wide range of eukaryote species.

### Model

In our model, we take into account four major factors that influence nucleosome assembly: DNA–histone interactions, nucleosome–nucleosome interactions, ATP-dependent nucleosome reorganization and the effect of barriers near TSSs. DNA, which is considered as a linear lattice of *N* base pairs, interacts with histone octamers in a sequence-dependent manner. In the model, each nucleosome is treated as a particle that occupies *k* = 147 bp of DNA. A nucleosome starting at 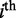 bp on DNA has an interaction energy *V_i_*. *V_i_* is computed from the model for *S. cerevisiae* nucleosome positioning of Kaplan *et al.* (1), as described in the ‘Materials and Methods’ section. Finally, nucleosomes act as ‘hard cores’; they are not permitted to overlap one another.

**Figure 1.**
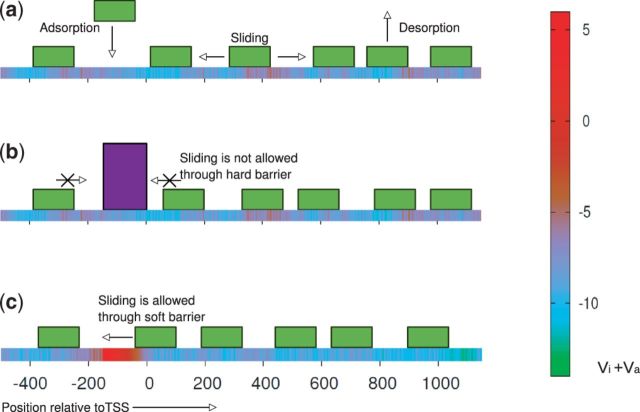
Schematic depiction of the model: (**a**) Three kinetic events: nucleosome binding, dissociation and sliding. The sequence dependent potential is represented by the color gradient as indicated in the sidebar. (**b**) A hard barrier (purple) prevents nucleosome binding and sliding through the TSS. (**c**) The soft barrier is the region with highly positive (red) sequence-dependent potential such that nucleosomes are repelled.

We have three kinetic events in our model (Figure 1a): (i) histone octamer binding to DNA to form nucleosomes (nucleosome adsorption); (ii) histone octamer release (nucleosome desorption); and (iii) lateral displacement of histone octamers along the DNA (nucleosome sliding). The actual deposition and dissociation of nucleosomes involve multiple steps. First, 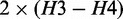 heterodimers are deposited by histone chaperones such as CAF1, and then 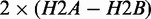 heterodimers are deposited by chaperones such as Nap1. During disassembly, 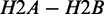 disassembles first, and only then the disassembly of 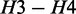 happens (24,25). However, for simplicity, in this article, we have approximated nucleosome binding and dissociation as single events where the octamer as a whole binds and dissociates at various locations along the DNA. Even though DNA–histone interactions in the presence of thermal forces can, in principle, result in all the three events discussed earlier in the text, thermally driven nucleosome desorption and sliding are far too slow to contribute to nucleosome positioning *in vivo* (17). It is known that ATP-dependent enzymes accelerate these processes (26–28).

In our model, we assume that, once a region of sufficiently long (at least *k* bp) empty DNA is available, nucleosome adsorption proceeds via an ATP-independent pathway, with a rate 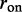. Adsorbed nucleosomes can dissociate in two ways – via thermal excitation or via the action of ATP-dependent enzymes. Thermally excited removal of a nucleosome depends on its location on the DNA: a nucleosome at position *i* is removed with rate 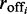,
(1)
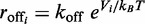

where 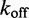 is the intrinsic removal rate per nucleosome. The ATP-independent adsorption and desorption rates must satisfy the Boltzmann condition
(2)
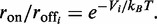



Given that 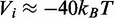, 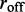 is bound to be extremely small. However, ATP-consuming remodeling enzymes greatly enhance the rate of nucleosome removal. The resulting net removal rate per nucleosome is given by
(3)
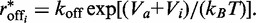



The enhancement of off-rate by the nucleosome-removing enzymes is accounted through a positive shift in the nucleosome binding potential by an amount *V_a_*, which can be considered to be the amount of chemical energy (from ATP hydrolysis) coupled into active nucleosome ‘eviction’ (see Supplementary Material for more details). For simplicity, we take *V_a_* to be constant; in principle, *V_a_* may vary with species or with chromosome location. Later in the text, we will specify the shift *V_a_* in terms of the average effective nucleosome binding free energy, 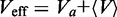, where 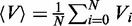.

The sliding of nucleosomes in our model is facilitated by ATP-dependent enzymes. We assume that remodelers slide nucleosomes at a rate 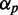 per nucleosome. During each sliding event, a nucleosome is moved in a randomly chosen direction until it collides with a neighbor. Based on experimental observation (27), we assume that this active pushing is independent of DNA sequence. Nucleosomes can also slide thermally as described in (17). However, thermal diffusion of nucleosomes is so slow that it has negligible influence on the yeast cell-cycle timescale (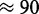 min) relevant here.

### Introduction of barriers to nucleosome occupancy

It has been observed that for most genes, adjacent to TSSs, there is a nucleosome free region (NFR). In this article, for each TSS considered, we introduce a 150-bp long barrier starting at 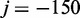 and ending at 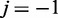, where *j* is sequence position relative to the TSS (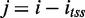). As the precise nature of the NFR is not known, we consider two possibilities:

(i) Hard barrier: A possibility is that the NFR could be caused by binding of non-histone proteins near TSSs (29). This could exclude nucleosome binding, as well as sliding through the TSS (Figure 1b). In this case, we model NFR as a hard-core barrier of length 150 bp. This also implies that 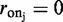 for 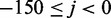.
(ii) Soft barrier: Another possibility is that the NFR is a result of a genomic sequence that disfavors nucleosome binding (6,30). However, with the help of remodeling machines, nucleosomes may slide over it, but with a potential energy cost that gradually rises with distance into the barrier region (Figure 1c). In this case, we model NFR as an energy barrier, where the binding energy *V_j_* is very large (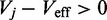). For the soft barrier, we take 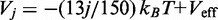, for 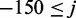 < 0. For all other values of *j*, *V_j_* is the sequence dependent potential described earlier. The slope (*m*) of the soft barrier is chosen to be −(13/150) *k_B_T* such that the occupancy near the barrier is comparable with the experimental data (see Supplementary Figure S1 in Supplementary Material).

## MATERIALS AND METHODS

We obtain the sequence dependent energy *V_i_* using the model of Kaplan *et al.* (1), who provide the static probability, *P_i_*, of finding a nucleosome starting at base pair *i* for any specified overall nucleosome density. The probability *P_i_* at low nucleosome density determines the potential 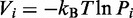, up to an overall constant.

We computed the dynamics of nucleosomes for this model, using continuous time stochastic simulations (31,32). In brief, at each step of the computation, we use the rates of the events which are possible, to compute the time interval until the next stochastically determined on-, off- or slide event. The event is then implemented, and the time is updated. Successive events are considered to be uncorrelated. ATP hydrolysis, while not explicitly included in the kinetic model, is implicitly included through the non-thermal remodeling (eviction and sliding) processes (17).

### Parameter values

Important parameters in the model are the nucleosome adsorption rate (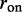), active nucleosome eviction rate (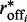) and the active nucleosome sliding rate (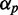). Numerical values of all these parameters are based on experimental data (15–17,32–34). Nucleosome assembly experiments in *Xenopus* egg extracts (15,32) determine 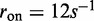 and the average value of *V_i_* under cellular conditions (15,16,32,33). The unknown constant in *V_i_* is fixed by the requirement that 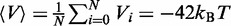. Given *V_i_*, the average density of nucleosomes determines 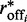. When 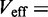 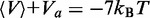, the nucleosome density is 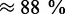, the approximate *in vivo* density in gene-rich regions. In this article, 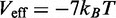, unless otherwise specified. The active sliding rate is fixed at 
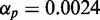
 s^−^^1^ (17,34), and the nucleosome size is also fixed at *k* = 147 bp.

## RESULTS AND DISCUSSION

We computed dynamics of nucleosomes for our model, starting at *t* = 0 from naked DNA, and allowing assembly of nucleosomes using the kinetic rules discussed earlier in the text. We show nucleosome occupancies at *t* = 1 h, unless specified otherwise, and all results are averaged over 1000 independent ‘replica’ runs (see Supplementary Figure S2). For comparison, in separate computations, we determined the thermal equilibrium nucleosome coverage probability, using the ‘Percus equation’ approach of (35).

### Nucleosome occupancy oscillations near a barrier require active chromatin remodeling

**Figure 2.**
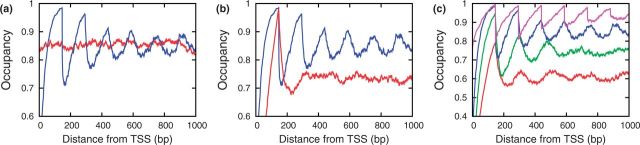
Effect of a hard barrier and ATPase activity. Nucleosome occupancy on a homogeneous DNA (**a**) in the presence (blue) and in the absence (red) of a barrier – ATPase activity here is specified through active sliding rate 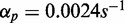 and active nucleosome removal parameter 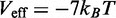; (**b**) in the presence of barrier but with (blue, 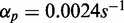, 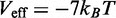) and without (red, 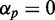, 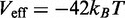) ATPase activity; (**c**) in the presence of barrier for different values of nucleosome removal – from bottom to top 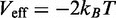 (red), 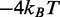 (green), 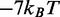 (blue), 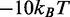 (pink).

We first discuss how the barrier and ATP-dependent remodeling machines work together to generate oscillatory nucleosome occupancy near TSSs. To begin with we consider nucleosome positioning without sequence effects, using a constant 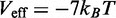. This gives us, as expected, a uniform occupancy along the DNA (Figure 2a, red curve). To determine how a barrier affects nucleosome positioning in this case, we repeat the simulations, but with a hard barrier at the TSS. Thus, the barrier induces a nucleosome positioning pattern, which is of ‘statistical’ origin, as there is no sequence dependence (Figure 2a, blue curve). However, unlike prior work (5,6,13), this nucleosome positioning pattern is appearing in <1 h for nucleosomes subject to ‘active non-thermal kinetics’ (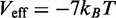, 
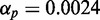
 s^−^^1^), i.e. active ATP-dependent chromatin remodeling.

Now, we ask what would happen on the same 1 h timescale in the ‘absence’ of non-thermal remodeling. To do this, we turn off the active sliding (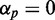) and set the off-rate to that appropriate for thermal equilibrium (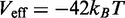); the result is a different pattern, with a nucleosome near the hard barrier, but with no longer-ranged occupancy oscillations (Figure 2b, red curve). A similar outcome resulted from use of soft barriers (see Supplementary Figure S3). The 1 h timescale is insufficient for nucleosome positional equilibration by thermal motions, and as a result, equilibration does not occur. Thus, ‘statistical’ positioning cannot occur on biologically relevant timescales via thermal motions of nucleosomes: active (ATP-driven) nucleosome remodeling is necessary, as observed experimentally by Zhang *et al.* (7). Statistical nucleosome positioning patterns observed in thermal equilibrium calculations are not experimentally relevant.

We can compute what the equilibrium nucleosome occupancy would be in thermal equilibrium for 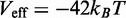: the result is essentially 100% occupancy with no oscillations (see Supplementary Figure S4). Reducing the nucleosome binding free energy to a non-physiological value does generate some oscillations (see Supplementary Figure S4) but with a shape unlike that is seen experimentally, with a succession of occupancy peaks near 100%. However, obtaining either of these equilibria would require thermalization on a gargantuan timescale far longer than the lifetime of any living organism. Thermal kinetics and therefore equilibrium statistical distributions are irrelevant to chromatin structure *in vivo*.

Returning to our 1 h kinetic simulations, Figure 2c shows how the nucleosome distributions vary with the amount of active nucleosome eviction, by varying 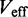 (holding the sliding rate fixed at 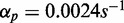). When nucleosome removals are rare, we observe a ‘saw tooth’-shaped occupancy (topmost pink curve). On the other hand, for very frequent removals, the oscillatory positioning pattern is suppressed (red curve) by suppression of the overall occupancy level, which reduces adjacent-nucleosome collisions and correlations. Positioning oscillations similar to those seen experimentally (7) are obtained for and intermediate rate of nucleosome removal (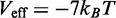, blue curve), which also yields a 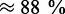 average coverage comparable with that found *in vivo*. We also varied the sliding rate 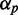 for a fixed nucleosome removal value 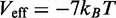 (see Supplementary Figure S5); the nucleosome profiles are much less sensitive to variation in the sliding rate than in the removal rate. We have also investigated how sliding affects nucleosome organization near TSS for small removal activity (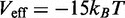). The results indicate that sliding alone cannot lead to statistical positioning observed *in vivo* (see Supplementary Figure S6).

### Sequence dominates over barrier effects beyond a characteristic sequence scale

**Figure 3.**
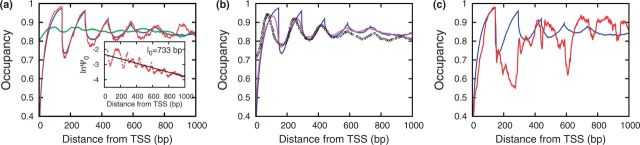
Effect of sequence: (**a**) Nucleosome occupancy in the presence of a hard barrier with homogeneous DNA (red), averaged over 100 genes in the presence of hard barrier (blue) and averaged over 100 genes in absence of any barrier (green). Inset: the gene-averaged deviation 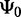 of profiles with and without barrier decays on a 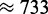 bp scale (see text). (**b**) Comparison of average occupancy data (blue: hard barrier; pink: soft barrier) with experimental data (open circles). (**c**) Nucleosome occupancy averaged over 100 genes with hard barrier (blue) and nucleosome occupancy of an individual gene with hard barrier (red; YGR034W, Chromosome VII). All results are for 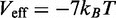 and 
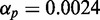
 s^−1^.

We now examine the effect of sequence-dependent nucleosome-DNA interactions on nucleosome occupancy near a barrier, in the presence of active nucleosome remodeling. We considered 100 different yeast genes (see Supplementary Material for details), and computed nucleosome organization over 10 000 bp regions around each TSS (see Supplementary Figure S7). First, we show nucleosome occupancy averaged over 100 genes (Figure 3a, blue curve) and on a homogeneous DNA (red), both in the presence of a hard barrier. This is compared with the occupancy with sequence effects but in the absence of any barrier (green). Close to the TSS, the occupancy with and without sequence (blue and red) effects are similar indicating that the effect of the barrier dominates there. However, as one moves away from the barrier, sequence becomes dominant.

To quantify the length over which barrier effects dominate over sequence, we computed the deviation function:
(4)
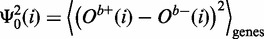



Here, 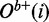 and 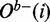 represent occupancy, at location *i*, with and without the hard barrier, respectively. 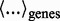 indicates averaging over gene sequences. We find that 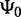 decreases exponentially, 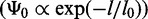, giving us a characteristic length 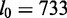 bp beyond which sequence dominates over the barrier effect (Figure 3a, inset). We also computed *l*_0_ with soft barrier (see Supplementary Figure S8), which is similar to the *l*_0_ with hard barrier.

Figure 3b compares the nucleosome occupancy averaged over 100 genes for hard (blue) and soft (pink) barriers, to experimental data (circles) (7). Both the amplitude and the phases of the oscillations produced by our model are in reasonable agreement with experimental data. The details of the shape of the oscillations generated by the soft barrier are closer to that seen experimentally than the more sharp shapes generated by the hard barrier, but this may well be due to measurement error or fluctuation in precise barrier position (36,37). Supplementary Figure S9 shows that changing remodeling activity can change nucleosome occupancy in ways similar to that seen in recent experiments (38).

In Figure 3, we have presented data averaged over 100 genes, similar to the gene-averaged presentation of occupancy in (7). However, we note that nucleosome occupancy along individual genes can strongly differ from the average. For some genes, the effect of sequence dominates even near the barrier; such a case is presented in Figure 3c for gene YGR034W (Chromosome VII). (Occupancies for other individual genes are shown in Supplementary Figure S10), where sequence can be seen to dominate over the average barrier effect after only 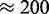 bp. Instead of computing *l*_0_ from a gene-averaged data, it is also possible to compute such a length scale for individual genes. We have done this for the 100 genes focused on in this study, with the result that the average correlation length is 731 bp, with a standard deviation (gene-to-gene variations) of 240 bp.

The aforementioned results show that ATP-dependent nucleosome removal and sliding activity combined with hard or soft barriers can result in *in vivo*-like oscillatory nucleosome occupancy downstream of NFRs. However, a set of *in vitro* experiments (39,40) find that in the presence of the remodelers ACF and RSC, there is no significant NFRs or adjacent oscillatory positioning. This suggests that creation of barriers (and therefore NFRs) requires something more than DNA sequence and remodelers like ACF and RSC. This raises an interesting question of the origin of barriers. As we saw in Supplementary Figure S1, the comparison of occupancy near a soft barrier with experimental data suggests that the shift in the positioning potential at NFR must be as high as 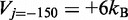
T. This is not likely to have its origin in DNA sequence, as no sequence is known to have such strong repulsion of nucleosomes. Therefore, the necessity of such a high barrier points to the occlusion of nucleosomes from NFR, perhaps by localized remodeling targeted by sequence-specific factors, to a degree that it requires us to use 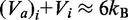
T at NFRs.

Apart from hard and soft barriers, there is a third possibility where a nucleosome itself can act as a barrier (41). We investigated this possibility and found that a firmly positioned nucleosome can also create statistical positioning pattern, with the help of active remodeling (see Supplementary Figure S11).

### Occupancy patterns near a barrier are established on the characteristic timescale for nucleosome eviction

**Figure 4.**
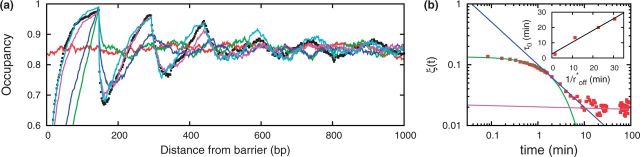
(**a**) Nucleosome occupancy along homogeneous DNA (uniform potential of −7 k_B_T) for different times after placing a hard barrier. Black dots represents steady state occupancy in presence of barrier. Just before placing the barrier (red), we see uniform occupancy. After 10 s (green), there is little positioning except immediately adjacent to the barrier. After 1 min (blue), positioning starts to appear. After 10 min (pink), the occupancy looks similar to the steady state occupancy (black dots), and after 30 min (cyan), the occupancy is nearly the same as the steady state occupancy. All curves are averaged over 1000 simulations. (**b**) Deviation of occupancy from its steady state value as a function of time (see text). Inset: The timescale over which positioning oscillations appear (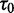) is essentially equal to the timescale for active nucleosome eviction (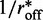).

If active remodeling is present, the 1 h timescales used in our calculations are sufficient to allow barrier- and sequence-controlled nucleosome occupancy patterns to appear. We now quantify in more detail the rate at which barrier-generated oscillations arise. To do this, we took a bare homogeneous DNA of 10 000 bp, and allowed (active) on, off and sliding kinetics to proceed for 30 min, in the absence of any barrier (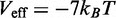, 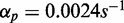). Then, we placed a hard barrier at the middle of the DNA and observed how the occupancy changed with time moving forward from barrier placement (see Figure 4a). Just before placing the barrier (red), the occupancy is uniform. After 10 s (green), one starts to see a positioning peak for the nucleosome immediately adjacent to the barrier, and then correlations build up further from the barrier (1 min, blue; 10 min, pink). After 10 min, little further change occurs (30 min, cyan) as the profile has reached that of the steady state (black dots).

To quantify the dynamics, we computed
(5)
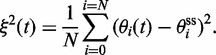

which measures how occupancy (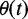) deviates from its steady state value (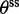) as a function of time after barrier placement. The result is shown in Figure 4b (red dots). Immediately after placing the barrier, the deviation decreases exponentially (green curve), 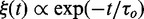, with a time constant 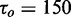 s. For times larger than 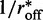, the deviation decreases via a short power law-like decay (blue curve), as the positioning profile approaches the steady state level (pink line). The timescale for the initial exponential relaxation is comparable with the timescale for nucleosome eviction, 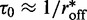, where 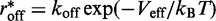. To test whether active eviction controls the exponential relaxation of the profile toward the steady state, we carried out the same simulation for different values of 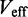 (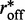). The result is that 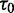 increases linearly (and is nearly equal to) the inverse of nucleosome eviction rate 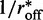 (Figure 4b inset). Thus, the main determinant of the time needed to establish nucleosome positioning patterns is the timescale for (active) nucleosome eviction.

### DNA site exposure kinetics near a barrier are dominated by sequence

**Figure 5.**
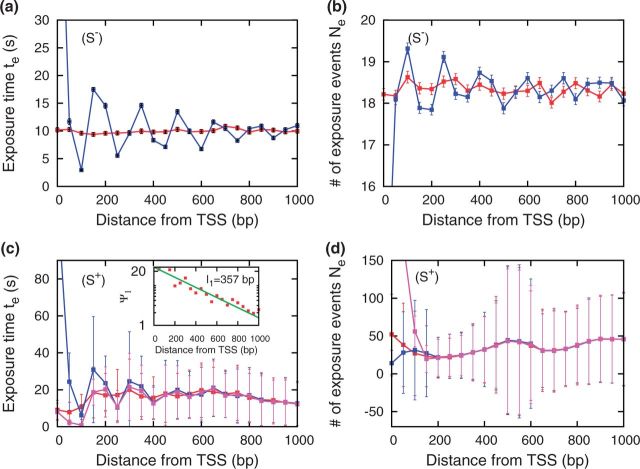
Average exposure time 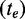 and total number of exposure events (*N_e_*) for different cases computed with (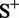) and without (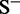) DNA sequence effects. (**a**) Average exposure time for homogeneous DNA in the presence (blue) and in the absence (red) of hard barrier. Bars represent standard error. (**b**) Number of exposure events for homogeneous DNA in the presence (blue) and in the absence (red) of a hard barrier. Bars represent standard error. (**c**) Exposure time, averaged over 100 gene sequences, in the presence of barrier (blue: hard barrier; pink: soft barrier) and in the absence (red) of barrier; Inset: The barrier versus non-barrier nucleosome distribution deviation 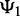 (see text) decreases exponentially as one moves away from the TSS. (**d**) Number of exposure events, averaged over 100 gene sequences, in the presence of barrier (blue: hard barrier; pink: soft barrier) and in the absence (red) of barrier. The bars in (c) and (d) represent standard deviation (not standard error) resulting from variability of 100 different gene sequences. 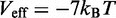 in all these cases.

As nucleosomes are evicted and slid, regions along the DNA are transiently exposed. This exposure likely influences binding of transcription factors. Here we examine how TSS barriers and DNA sequence influence site-exposure kinetics. We consider a series of 10 bp-long sites near the barrier; when all 10 bp are accessible (not covered by any nucleosome), we consider the site to be ‘exposed’. As nucleosomes move, exposure events for a given site begin and then end. In a simulation of a 1 h period, we compute the total number of exposure events (*N_e_*) and their average durations of exposure (*t_e_*) that occur during the last 30 min; (see Figure 5; 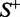 represents quantities computed including sequence effects and 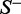 represents the same without sequence effects).

To understand barrier effects, we first computed *t_e_* and *N_e_* with a hard barrier and without any barrier, along a homogeneous DNA (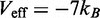 T). In the absence of any barrier, both average exposure time and total number of exposure events are independent of sequence position (red curves in Figure 5a and b). Presence of the barrier changes average exposure time significantly and introduces positional oscillations in it (Figure 5a, blue curve). Comparing with the occupancy for the corresponding case (Figure 2a, blue curve), close to the barrier, there is an inverse relation between occupancy and average exposure time–when occupancy is maximum, exposure time is minimum. The barrier also introduces oscillations in the total number of exposure events, but with a much weaker amplitude relative to the mean (Figure 5b, blue curve) than for *t_e_*.

To investigate how DNA sequence influences the exposure time and number of exposure events, we carried out similar simulations with sequence (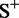) for our set of 100 genes. The results averaged over the 100 genes are shown in Figure 5c and d. Figure 5c shows average exposure time varies along DNA for hard and soft barriers (blue: hard barrier; pink: soft barrier) and without any barrier (red). When there is no barrier, exposure time is nearly uniform except immediately adjacent to the TSS. The presence of the barrier introduces oscillations in *t_e_*; however, not too far from the barrier, sequence starts to dominate over the barrier, and the *t_e_* in the presence and absence of the barrier becomes equal. Similar to the calculation of 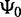 mentioned previously, we measured the distance over which barrier effects dominate over sequence using the deviation
(6)
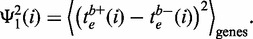



Here 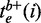 and 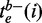 represent average exposure time, at location *i*, with and without barrier, respectively. We find that 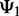 decreases exponentially 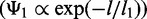, giving us the length 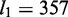 bp, beyond which sequence effects dominate (Figure 5c, inset, hard barrier case). Comparing this with the corresponding case in Figure 3a, we find that 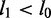. This reveals an interesting feature: sequence effects have a stronger influence on kinetic quantities (e.g. exposure time) than on occupancy.

Results of similar calculations for the number of exposure events (*N_e_*, Figure 5d) show total numbers of exposure events, averaged over sequence, with (blue) and without (red) barrier. The sequence, in the absence of barrier, introduces an overall shape for *N_e_*. This can be tracked back to the shape of the potential (Supplementary Figure S12 shows the potential averaged over 100 genes). This shape was not seen in the occupancy or in the profile for *t_e_*; apparently *N_e_* is more sensitive to sequence. When we introduce the barrier, very close to the TSS, the barrier has some effect. However, as we go away from the TSS, the barrier effect disappears quickly; only 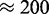 bp from the TSS, the barrier has lost its influence. Thus, sequence has a major effect in determining number of exposure events *N_e_*.

The error bars in Figure 5c and d represent the standard deviation obtained from averaging over 100 different genes. The large deviation indicates that *t_e_* and *N_e_* for individual genes can strongly vary from the average behavior. To demonstrate this, Supplementary Figure S13 shows *t_e_* and *N_e_* for two individual genes. Both *t_e_* and *N_e_* show strong deviations from the average, with *N_e_* showing a peak towards the end of the gene; also, the barrier has little effect on both *t_e_* and *N_e_*.

Our results, overall, indicate that kinetic quantities such as the average exposure time and the number of exposure events are much more sensitive to sequence when compared with occupancy. To examine this further, we computed how the average density and number of exposure events vary with potential energy. We computed 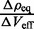 and 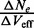 (see Supplementary Material and Supplementary Figure S14). The results show that, unlike nucleosome density, the number of exposure events is highly sensitive to change in potential energy. A 1 *k_B_*T change in potential energy can induce a 100% change in the number of exposure events, whereas the average density change is only 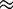 2%. Therefore, even if the average occupancy at two different locations in a genome is similar, the nucleosome dynamics at these two locations can be very different. A 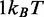 change in potential energy is not likely to change the average nucleosome coverage, but the same potential energy change can significantly alter the corresponding nucleosome dynamics, and therefore the rate at which gene-regulating factors can bind.

## CONCLUSION

In this article, we have used mathematical modeling to investigate how nucleosome-depleting barriers, DNA sequence and ATP-dependent molecular machines work together to control nucleosome positioning. Our model provides insight into why ATP-dependent remodeling is absolutely necessary to obtain the oscillatory statistical positioning observed near nucleosome-depleting barriers; without active nucleosome removal, equilibration of nucleosome positions is far too slow to occur on experimental timescales. Our results suggest that ATP-dependent nucleosome eviction is crucial in obtaining the statistical positioning.

Our model also provides insight into the role of DNA sequence in determining nucleosome kinetics and positioning near TSSs. We find that nucleosome occupancy for individual genes, even in the presence of barrier, can be highly sequence dependent. However, when averaged over many genes, the occupancy appears similar to one computed with homogeneous sequence. As all the earlier models that neglected sequence effects could reproduce experimentally observed, but gene-averaged, occupancy, it has been argued in the literature that the barrier is what sets the nucleosome occupancy near TSSs. Our work suggests that this may not always be true; for some genes, sequence can drive strong positioning of nucleosomes near to TSSs that may affect regulation of those genes.

Using the model, we have computed a number of important kinetic quantities. First, we have obtained the timescale over which the statistical positioning builds up. We find that the timescale for active nucleosome removal is essentially the timescale for establishment of steady-state positioning patterns. Then, we have computed the mean time for which a given 10-bp region near the barrier remains exposed, and the number of such exposure events during the course of our simulation. We find that, unlike steady-state occupancy, site exposure kinetics near a barrier, even after averaging over many genes, is highly influenced by the DNA sequence. Thus, kinetics of site exposure can show sensitivity to sequence that is not apparent in nucleosome occupation profiles; it would be extremely useful to develop a method for measurement of site exposure statistics along chromatin. In short, in this article, we have shown that the nucleosome kinetics near TSSs are determined not just by the presence of a barrier but through a complex interplay between ATP-dependent chromatin remodeling, DNA sequence and the barrier effects. However, one needs further study to probe how exactly the kinetics would alter if one considers detailed dynamics of H2A-H2B heterodimers and H3-H4 tetramer.

Without question, there are aspects of *in vivo* nucleosome positioning that arise from factors other than those considered here, namely, barriers at NFRs, DNA sequence and remodeling activities. As an example, we note the asymmetry between nucleosome occupation upstream and downstream of NFRs, which may be generated by occlusion of nucleosomes in the promoter region by transcription factors (which may bind in patterns that vary from cell to cell as well as from gene to gene), or by RNA polymerase activity in the gene itself. There may also simply be different remodeling activities upstream and downstream of TSSs. Although our present model does not explain this asymmetry, it does indicate clearly the need for an NFR-generating mechanism (our ‘barrier’), plus active nucleosome remodeling, the latter necessary for establishment of nucleosome positioning patterns during timescales less than a cell cycle period.

## SUPPLEMENTARY DATA

Supplementary Data are available at NAR Online, including [42–44].

## FUNDING

At Northwestern University, NSF Grants [MCB-1022117 and DMR-1206868]; NIH-NCI grant [U54CA143869-01]. At IIT Bombay, CSIR grant No. [37 (1582)13/EMR-II to R.P.], and a fellowship from UGC, India (to J.P.). Funding for open access charge: Grants from Council of Scientific & Industrial Research, India and funding from Indian Institute of Technology Bombay.

*Conflict of interest statement*. None declared.

## Supplementary Material

Supplementary Data
