# Supplementary material for: Nucleosome positioning and kinetics near transcription-start-site barriers are controlled by interplay between active remodeling and DNA sequence
Source: Nucleic Acids Res. 2013 Sep 24;42(1):128–36. doi: 10.1093/nar/gkt854 (PMC3874171; doi:10.1093/nar/gkt854)
Supplement: Supplementary Data [file supp_42_1_128__index.html]

Nucleosome positioning and kinetics near transcription-start-site barriers are controlled by interplay between active remodeling and DNA sequence — Nucleosome positioning and kinetics near transcription-start-site barriers are controlled by interplay between active remodeling and DNA sequence — Supplementary Data 

# Nucleosome positioning and kinetics near transcription-start-site barriers are controlled by interplay between active remodeling and DNA sequence

## Supplementary Data

files

**Files in this Data Supplement:**

- Supplementary Data - pdf file
