# Supplementary material for: Nucleosome positioning and kinetics near transcription-start-site barriers are controlled by interplay between active remodeling and DNA sequence
Source: Nucleic Acids Res. 2013 Sep 24;42(1):128–36. doi: 10.1093/nar/gkt854 (PMC3874171; doi:10.1093/nar/gkt854)
Supplement: Supplementary Data [file supp_gkt854_nar-01176-m-2013-File019.pdf]

SUPPLEMENTARY MATERIALS

Nucleosome positioning and kinetics near transcription-start-site barriers are controlled by interplay between active remodeling and DNA sequence

Jyotsana J. Parmar, John F. Marko and Ranjith Padinhateeri

Active nucleosome removal

We follow Kramers rate theory [1] to incorporate ATP-dependent activity, expressing the rates as:

$$r^*_{\text{off}_i} = (k_{\text{off}} + k_a e^{\Delta\mu}) e^{V_i} \quad (1)$$

$$= k_{\text{off}} \left(1 + \frac{k_a}{k_{\text{off}}} e^{\Delta\mu}\right) e^{V_i} \quad (2)$$

$$= k_{\text{off}}\ e^{V_a} e^{V_i} \quad (3)$$

where $k_{\text{off}} e^{V_i}$ is the thermal removal rate, $k_a e^{\Delta\mu}$ is the ATP-dependent active contribution, and $V_a = \ln\left(1 + \frac{k_a}{k_{\text{off}}} e^{\Delta\mu}\right)$. We have taken V_a to be a constant. This is an idealization as the active contribution depends on ATP/ADP concentration ($\Delta\mu$ is a function of ATP and ADP concentration) as well as on the concentration of the remodellers and their specificity (represented by k_a).

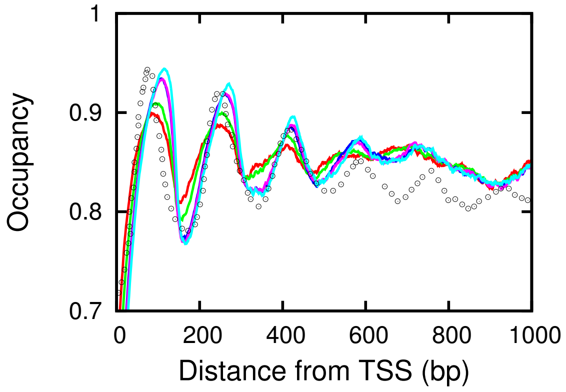

Fig. S1: Nucleosome occupancy next to a soft barrier. Each curve is computed by changing the slope (m) of the potential that forms the barrier. Red: m= -5/150, Green: m= -7/150, Blue: m= -12/150, Pink: m= -13/150, Cyan: m= -17/150. Black circles are the experimental data [2]. In our simulations (presented in main text) we have taken m= -13/150 (soft barrier corresponding to the pink curve). All the curves here are with $V_{\rm eff} = -7k_BT$ and $\alpha_p = 0.0024s^{-1}$.

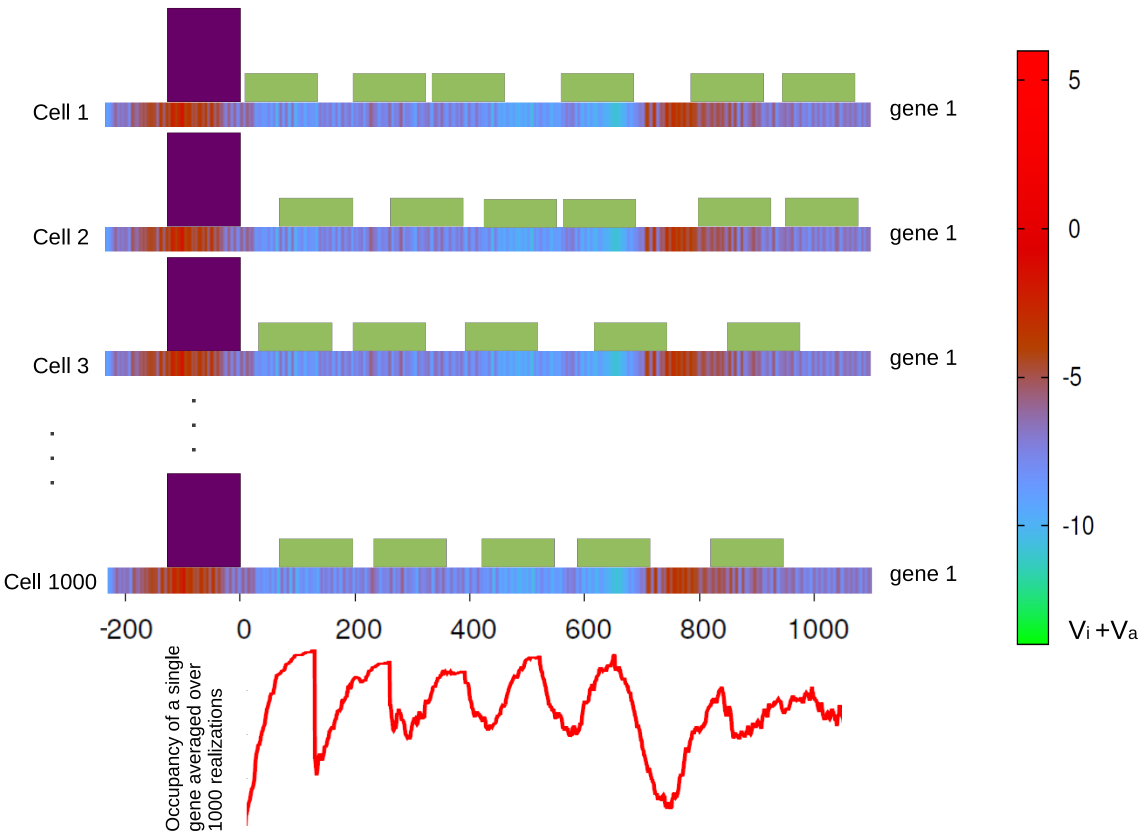

Fig. S 2: Schematic depiction of our "replica" averaging: Average occupancy on a given region (e.g., gene 1), is computed by performing 1000 simulations, with all parameters being the same. The color gradient on the lattice represents DNA-histone binding potential, where red corresponds to more repulsive sequence. Green blocks represent nucleosomes and the purple block represents the hard physical barrier.

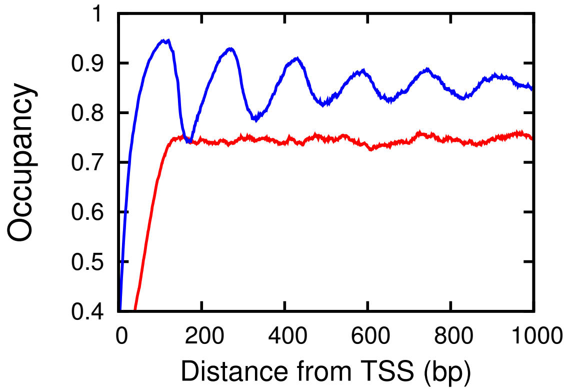

Fig. S 3: Nucleosome occupancy in presence of a soft barrier on a homogeneous DNA with remodeling activity (blue) ($V_{\text{eff}} = -7k_BT$ and $\alpha_p = 0.0024s^{-1}$) and without any remodeling activity (red) ($V_{\text{eff}} = -42k_BT$ and $\alpha_p = 0$).

Nucleosome positioning at thermal equilibrium

To calculate nucleosome positioning at thermal equilibrium, we used the model from Ref. [3]. In this model, the N-nucleosome energy is given by

$$H = \sum_{i=1}^{N} U(n_{i+1} - n_i) + \sum_{i=1}^{N} V_{n_i} \tag{4}$$

where n_i is the starting position of ith nucleosome, $U(m)$ is the nucleosome hard-core interaction potential and V_n is the sequence-dependent binding potential energy. Based on the calculations by Percus [4], the equilibrium probability (p_i) of finding a nucleosome starting at location i satisfies the relation [3]

$$h_i = p_i \frac{1}{\left(1 - \sum_{j=1}^{k-1} p_{i-j}\right)} \tag{5}$$

where, $h_n = \frac{H_n e^{\beta(\mu - V_n)}}{1 + H_n e^{\beta(\mu - V_n)}}$, and, $H_n = \prod_{m=2}^{k} (1 - h_{n+m-1})$; here μ is the chemical potential, equivalent to our ATP-dependent energy V_a and k is the size of the nucleosome. In our calculation, we take $\langle \mu - V_n \rangle = -V_{\text{eff}}$.

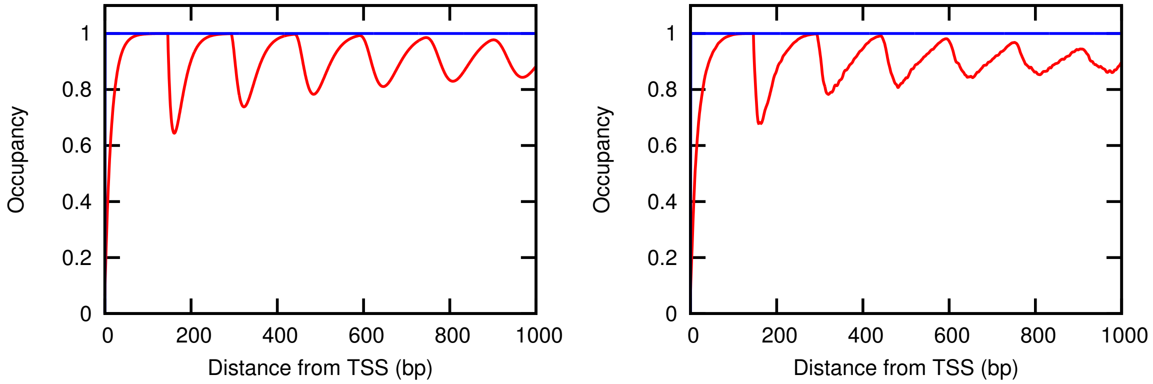

Fig. S 4: Nucleosome occupancy based on equilibrium statistical mechanics computation using Percus equation for homogeneous DNA (left) and for average of 100 genes (right). Blue: occupancy for parameters corresponding to non-ATP conditions: ($V_{\text{eff}} = -42k_BT$). Since purely thermal events, at physiological temperature, cannot lead to any significant nucleosome disassembly, the resulting occupancy is nearly 1 everywhere. Red: Parameters are chosen such a way ($V_{\text{eff}} = -7k_BT$) that the average occupancy is around 88%.

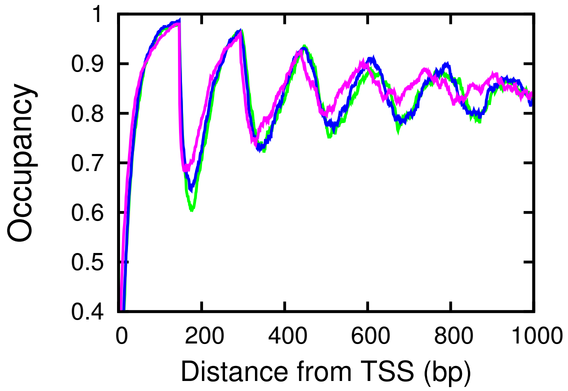

Fig. S5: Nucleosome occupancy on a homogeneous DNA in presence of a hard barrier with different sliding rates: Green: $\alpha_p = 0.000024s^{-1}$, Blue: $\alpha_p = 0.00024s^{-1}$, and pink: $\alpha_p = 0.0024s^{-1}$. Nucleosome removal rate is the same for all the curves ($V_{\text{eff}} = -7k_BT$). Note that the change in sliding rate does not affect the overall occupancy pattern significantly.

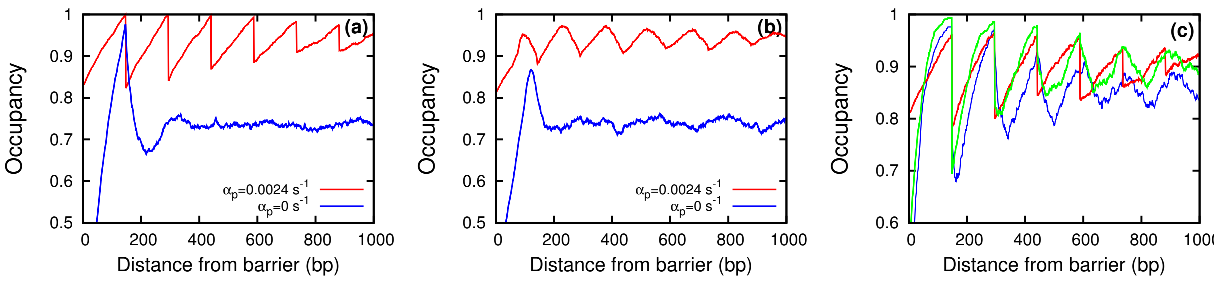

Fig. S6: Effect of sliding on nucleosome occupancy (with homogeneous sequence) in the presence of hard barrier (a), and soft barrier (b). Red curves in (a) and (b) show occupancy with parameters $\alpha_p = 0.0024s^{-1}$ and $V_{\text{eff}} = -15k_BT$, whereas blue curves show occupancy with $\alpha_p = 0$ and $V_{\text{eff}} = -15k_BT$. This shows that, as far as statistical positioning is concerned, sliding becomes important when the removal activity is small ($V_{\text{eff}} = -15k_BT$). However, low removal activity would lead to very high density of nucleosomes. In (c), we stop the binding and dissociation events, once the density is reached 88%. After reaching the density of 88%, simulation is run for one hour, for $\alpha_p = 0.0024\ s^{-1}$ (red) and $\alpha_p = 0.00024\ s^{-1}$(green). Then we compare this with our normal occupancy pattern (blue, with active sliding and disassembly throughout the simulation with $V_{\text{eff}} = -7k_BT$ and $\alpha_p = 0.0024\ s^{-1}$).

Gene regions considered in this study:

In this paper, we study nucleosome organization in the ORF regions of 100 yeast genes (NCBI database) with known transcription start sites (TSS) [5]. As per Ref. [5], the 100 top verified genes with known TSS were chosen. These genes are highly expressed genes having >10 mRNA copies per cell. We simulated nucleosome kinetics in all these 100 gene regions, and computed occupancy and site exposure kinetics as described in the main text. Below we list all the 100 genes used in this study.

No.	Chromosome	ORF
1	III	YCR012W
2	VIII	YHR021C
3	XII	YLR110C
4	XVI	YPL079W
5	XII	YLR075W
6	IX	YIL018W
7	VII	YGR118W
8	XIV	YNL178W
9	XII	YLR167W
10	IV	YDR382W
11	XV	YOR182C
12	I	YAL038W
13	XV	YOL086C
14	V	YER117W
15	VII	YGL031C
16	VIII	YHR010W
17	VII	YGL123W
18	XVI	YPR080W
19	II	YBR118W
20	XV	YOL039W
21	V	YER102W
22	X	YJL189W

No.	Chromosome	ORF
23	VIII	YHR203C
24	XV	YOL121C
25	II	YBR010W
26	XVI	YPL143W
27	IV	YDL075W
28	VII	YGR034W
29	II	YBR031W
30	IV	YDL229W
31	XIII	YMR116C
32	XII	YLR044C
33	XIV	YNL302C
34	XVI	YPR043W
35	XVI	YPR132W
36	IV	YDL191W
37	IV	YDL130W
38	XV	YOR293W
39	XV	YOL109W
40	X	YJL190C
41	VII	YGL147C
42	VII	YGR192C
43	XIV	YNL031C
44	X	YJR123W
45	II	YBL092W
46	XVI	YPL131W
47	XV	YOR063W
48	VII	YGL030W
49	XV	YOR234C
50	XIII	YML024W
51	XVI	YNL145W
52	XV	YOR369C
53	IX	YIL052C

No.	Chromosome	ORF
54	IV	YDR447C
55	IV	YDL081C
56	X	YJR009C
57	II	YBR181C
58	IV	YDR450W
59	III	YCR031C
60	XV	YOR167C
61	XII	YLR441C
62	VI	YFR031C
63	XV	YOL040C
64	VII	YGL135W
65	VIII	YHR174W
66	VII	YGR148C
67	II	YBL087C
68	IV	YDR050C
69	VII	YGR085C
70	XI	YKL152C
71	IV	YDL136W
72	XII	YLR185W
73	XIV	YNL162W
74	XV	YOL120C
75	IV	YDL083C
76	X	YJR094W
77	XI	YKL180W
78	XV	YOR312C
79	II	YBR189W
80	X	YJR145C
81	V	YEL034W
82	XIII	YML028W
83	IV	YDR012W
84	XII	YLR340W

No.	Chromosome	ORF
85	II	YBL003C
86	VIII	YHR141C
87	XII	YLR029C
88	XII	YLR406C
89	XII	YLR300W
90	II	YBL027W
91	IV	YDR033W
92	XII	YLL045C
93	IV	YDL061C
94	IV	YDR471W
95	XVI	YPL037C
96	IV	YDR276C
97	XIV	YNL135C
98	IV	YDR224C
99	XII	YLR333C
100	VII	YGR209C

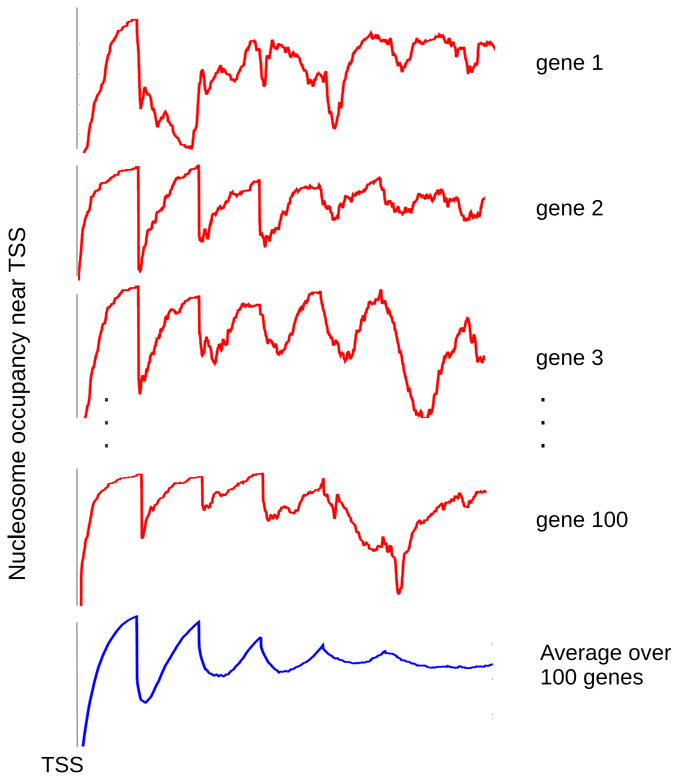

Fig. S 7: Procedure to compute gene-averaged nucleosome occupancy: First, occupancies for individual genes are generated as shown in Fig. S2 (red curves). Then these curves are averaged to obtain the gene-averaged occupancy (blue curve).

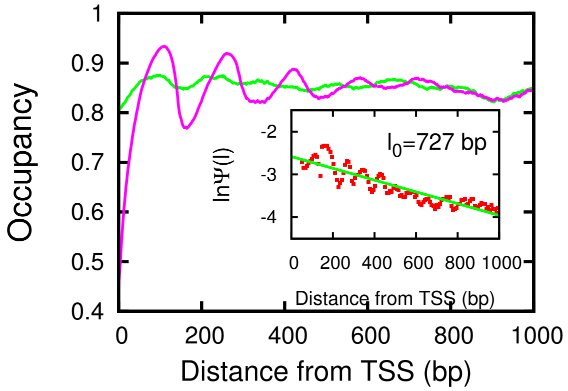

Fig. S8: Nucleosome occupancy averaged over 100 genes ($V_{\text{eff}} = -7k_BT$ and $\alpha_p = 0.0024s^{-1}$) in the presence of a soft barrier (pink), and in the absence of any barrier (green). Inset: the gene-averaged deviation of profiles with and without soft barrier decays on a ≈ 727 bp, very similar to the result from the hard barrier (≈ 733 bp).

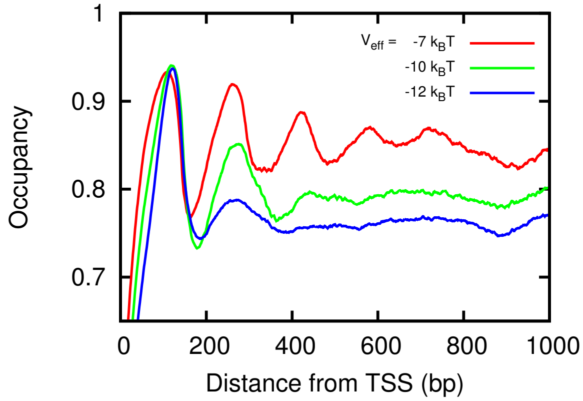

Fig. S9: Effect of remodeling on nucleosome occupancy (soft barrier). Red curve represents: $V_{\text{eff}} = -7k_BT$ and $\alpha = 0.0024\ s^{-1}$; green curve is with lesser activity, i.e. ($V_{\text{eff}} = -10k_BT$ and no sliding), and blue curve is with even lesser activity i.e. ($V_{\text{eff}} = -12k_BT$ and no sliding). The shape of the occupancy profiles here are qualitatively similar to the ones observed by Gkikopoulos et al [6], when a set of major remodellers are deleted/disrupted. The simulation results here are averaged over 100 genes.

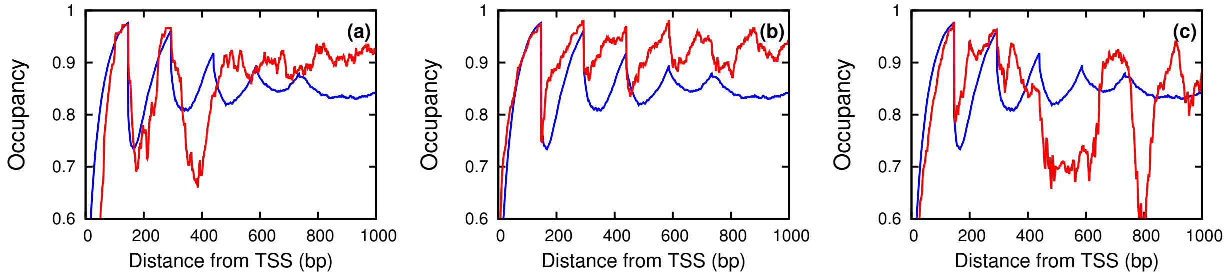

Fig. S 10: Nucleosome occupancy near a hard barrier for three individual genes (a) YIL018W, chromosome IX, (b) YCR012W, chromosome III, (c) YDR382W, chromosome IV (red curves in each plot) compared with the occupancy averaged over 100 genes (blue curves). For all the three plots, $V_{\text{eff}} = -7k_BT$ and $\alpha_p = 0.0024s^{-1}$.

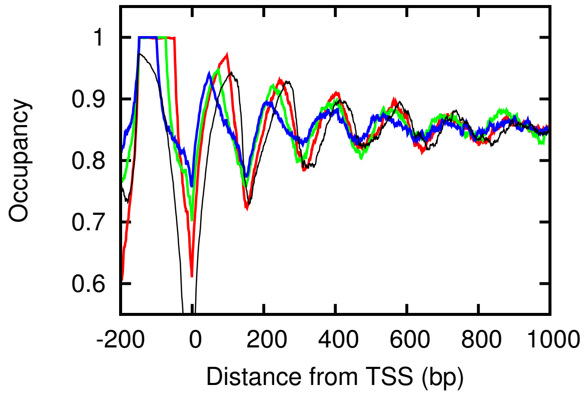

Fig. S 11: Nucleosome occupancy with a strongly absorbing TSS (sink): First time, when a nucleosome comes into a specified region (region between j=-147 and j=-147-x), it gets absorbed there (like a sink) permanently. Nucleosome occupancy for x=50 bp (red), x=74 bp (green) and x=100 bp (blue); occupancy in presence of a soft barrier (black). This shows that a nucleosome sink can also cause statistical positioning. For all the curves, $\alpha_p = 0.0024s^{-1}$ and $V_{\text{eff}} = -7k_BT$. j is the distance from TSS in bp.

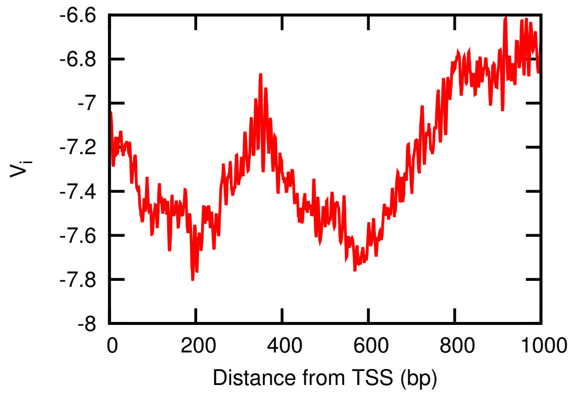

Fig. S12: Sequence dependent potential averaged over 100 genes near TSS. Note that this shape of the potential is reflected in the number of exposure events in the main text.

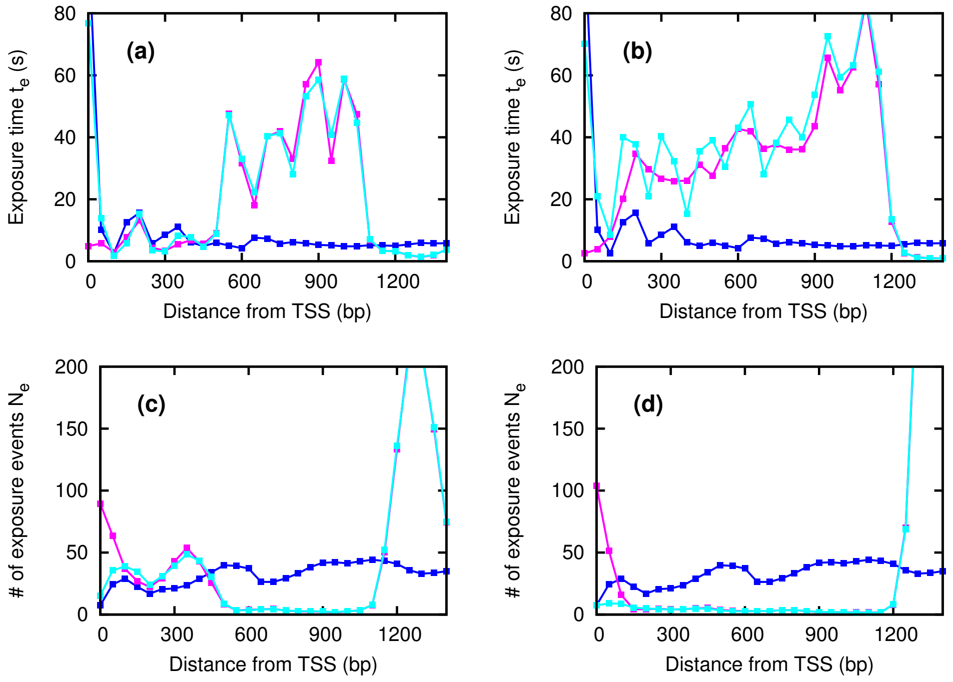

Fig. S13: Average exposure time (t_e) and number of exposure events N_e for two individual genes. The left figures (a) and (c) are for gene YIL018W (Chromosome IX) and right figures (b) and (d) are for the gene YCR012W (Chromosome III). In all the figures, blue: behavior near a hard barrier averaged over 100 genes; cyan: for individual gene in the presence of a hard barrier; pink: for individual gene in the absence of any barrier. In all the plots, $V_{\text{eff}} = -7k_BT$ and $\alpha_p = 0.0024s^{-1}$.

Number of exposure event is highly sensitive to small change in potential

To examine how a small change in potential influences the nucleosome coverage and number of exposure events, we computed derivatives (finite difference) of average equilibrium density (ρ_{eq}) and number of exposure events (N_e) with respect to potential (V_{eff}) as follows:

$$\gamma_\rho = \frac{\Delta \rho_{eq}}{\Delta V_{\text{eff}}} \tag{6}$$

$$\gamma_N = \frac{\Delta N_e}{\Delta V_{\text{eff}}} \tag{7}$$

These are computed from simulation data on homogeneous sequences with various V_{eff}. The results presented in Fig. S14 (left) show that change in number of exposure events is much higher than the change in density. To convert it to a percentage change, we computed:

$$\phi_\rho = \frac{1}{\rho_{\text{eq}}} \frac{\Delta \rho_{eq}}{\Delta V_{\text{eff}}} \times 100 \tag{8}$$

$$\phi_N = \frac{1}{N_e} \frac{\Delta N_e}{\Delta V_{\text{eff}}} \times 100 \tag{9}$$

These quantities plotted in Fig. S14 (right) show that a $1k_B$T change in potential energy can induce a 100% change in the number of exposure events, while the average density change is only $\approx 2\%$.

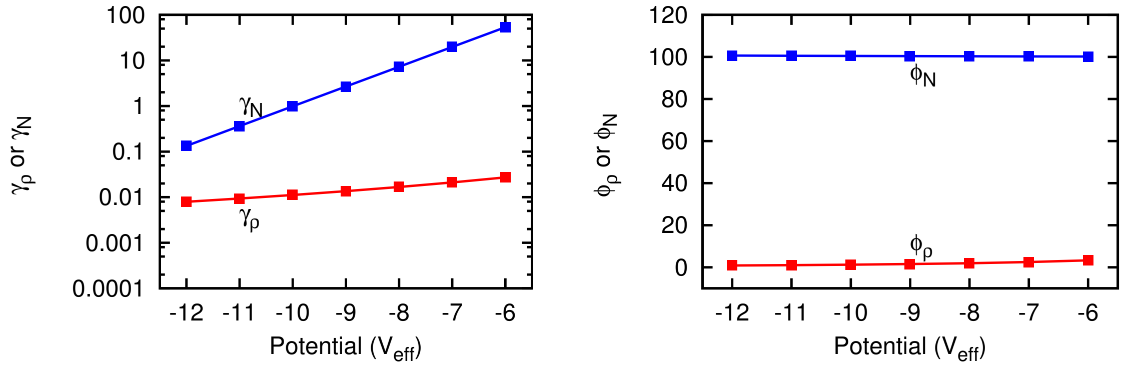

Fig. S14: (left): Equilibrium density-change when the effective potential is changed ($\gamma_\rho = \frac{\Delta \rho_{eq}}{\Delta V_{\text{eff}}}$); number of exposure events-change when the effective potential is changed ($\gamma_N = \frac{\Delta N_e}{\Delta V_{\text{eff}}}$). (right) The same quantities expressed as percentage change: percentage change in equilibrium density ($\phi_\rho = \frac{1}{\rho_{\text{eq}}} \frac{\Delta \rho_{eq}}{\Delta V_{\text{eff}}} \times 100$); percentage change in number of exposure events ($\phi_N = \frac{1}{N_e} \frac{\Delta N_e}{\Delta V_{\text{eff}}} \times 100$).

[1] Kafri Y, Lubensky DK, Nelson DR (2004) Dynamics of Molecular Motors and Polymer Translocation with Sequence Heterogeneity. *Biophys. J.* 86:3373–3391.

[2] Zhang Z, et al. (2011) A Packing Mechanism for Nucleosome Organization Reconstituted Across a Eukaryotic Genome. *Science* 332:977–980.

[3] Schwab DJ, Bruinsma RF, Rudnick J, Widom J (2008) Nucleosome Switches. *Phys. Rev. Lett.* 100:228105.

[4] Percus JK (1976) Model for density variation at a fluid surface. *J. Stat. Phys.* 15:423–435.

[5] Zhang Z, Dietrich FS (2005) Mapping of transcription start sites in Saccharomyces cerevisiae using 5' SAGE. *Nucleic Acids Res.* 33:2838–2851.

[6] Gkikopoulos T, et al. (2011) A role for Snf2-related nucleosome-spacing enzymes in genome-wide nucleosome organization. *Science* 333:1758–1760.
